# Exploring the Metabolism of Loxoprofen in Liver Microsomes: The Role of Cytochrome P450 and UDP-Glucuronosyltransferase in Its Biotransformation

**DOI:** 10.3390/pharmaceutics10030112

**Published:** 2018-08-02

**Authors:** Riya Shrestha, Pil Joung Cho, Sanjita Paudel, Aarajana Shrestha, Mi Jeong Kang, Tae Cheon Jeong, Eung-Seok Lee, Sangkyu Lee

**Affiliations:** 1 BK21 Plus KNU Multi-Omics based Creative Drug Research Team, College of Pharmacy, Research Institute of Pharmaceutical Sciences, Kyungpook National University, Daegu 41566, Korea; riya.shrestha07@gmail.com (R.S.); whvlfwjd@naver.com (P.J.C.); sanjitapdl99@gmail.com (S.P.); 2 College of Pharmacy, Yeungnam University, Gyeongsan 38541, Korea; aarajanashrestha1@gmail.com (A.S.); jkang@ynu.ac.kr (M.J.K.); taecheon@ynu.ac.kr (T.C.J.); eslee@ynu.ac.kr (E.-S.L.)

**Keywords:** loxoprofen, CYP, UGT, human liver microsomes, LC-HR/MS

## Abstract

Loxoprofen, a propionic acid derivative, non-steroidal anti-inflammatory drug (NSAID) is a prodrug that is reduced to its active metabolite, trans-alcohol form (Trans-OH) by carbonyl reductase enzyme in the liver. Previous studies demonstrated the hydroxylation and glucuronidation of loxoprofen. However, the specific enzymes catalyzing its metabolism have yet to be identified. In the present study, we investigated metabolic enzymes, such as cytochrome P450 (CYP) and UDP-glucuronosyltransferase (UGT), which are involved in the metabolism of loxoprofen. Eight microsomal metabolites of loxoprofen were identified, including two alcohol metabolites (M1 and M2), two mono-hydroxylated metabolites (M3 and M4), and four glucuronide conjugates (M5, M6, M7, and M8). Based on the results for the formation of metabolites when incubated in dexamethasone-induced microsomes, incubation with ketoconazole, and human recombinant cDNA-expressed cytochrome P450s, we identified CYP3A4 and CYP3A5 as the major CYP isoforms involved in the hydroxylation of loxoprofen (M3 and M4). Moreover, we identified that UGT2B7 is the major UGT isoform catalyzing the glucuronidation of loxoprofen and its alcoholic metabolites. Further experimental studies should be carried out to determine the potency and toxicity of these identified metabolites of loxoprofen, in order to fully understand of mechanism of loxoprofen toxicity.

## 1. Introduction

**Figure 1:**
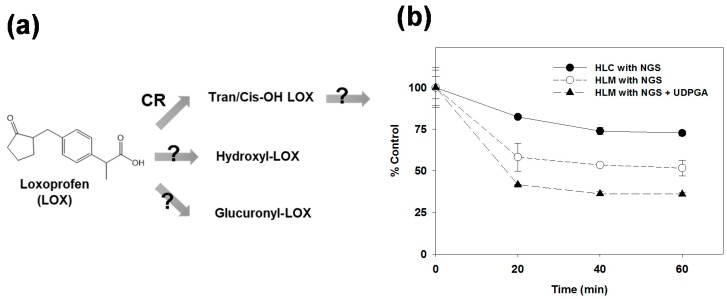
Chemical structure of loxoprofen and its metabolites (**a**). Metabolic stability of 5 µM of loxoprofen in 0.25 mg/mL of human liver cytosols (HLC) in the presence of β-reduced nicotinamide adenine dinucleotide phosphate (β-NADPH) regeneration system (NGS) and 0.25 mg/mL of human liver microsomes (HLMs) in the presence of NGS and uridine 5′-diphosphoglucuronic acid trisodium (UDPGA) incubated at 37 °C for 60 min (**b**). The data are represented as mean ± standard error (S.E) of the triplicate samples.

Loxoprofen, 2-(4-((2-Oxocyclopentyl)methyl)phenyl) propionic acid, is a non-selective non-steroidal anti-inflammatory drug (NSAID) developed in Japan by Daiichi Sankyo Co. Ltd. in 1986 [1]. Loxoprofen is mainly used to treat pain and inflammation related to musculoskeletal and joint disorders, such as rheumatoid arthritis, osteoarthritis [2,3], and post-operative pain [1]. Loxoprofen is a prodrug metabolized in the liver by carbonyl reductase enzyme to its active trans-alcohol metabolite (Trans-OH), 2-(4-((trans-2-hydroxycyclopentyl)-methyl)-phenyl) propionic acid (Figure 1a) [1,4]. The active metabolite exhibits anti-inflammatory activity by inhibiting the cyclooxygenase enzymes, thus impairing the formation of the chemical prostaglandin, which is responsible for pain and inflammation [5,6]. Although it has been reported to be safer than its counterparts and is one of the most prescribed anti-inflammatory drugs in Japan, loxoprofen has shown diverse adverse drug reactions (ADRs) and has recorded the highest rate of adverse effects in Korea in the first half of 2017 [7,8,9,10]. Some of the ADRs of loxoprofen are gastro-intestinal disorder, nausea, hypersensitivity, blood disorders (leukopenia and hemolytic anemia), renal disorders, drug induced liver injury, shock, and anaphylaxis [1]. In addition, the potential drug-drug interaction of loxoprofen with aspirin, enoxacin, and valacyclovir has been reported [10,11,12]. Thus, the ADRs of loxoprofen might be attributed to its drug-drug interactions with other marketed drugs. 

The reaction phenotyping of a drug is an essential part of the drug discovery and development process, which helps in preventing the ADRs derived from drug–drug interactions. While the pharmacokinetic profiling of loxoprofen and its active metabolites has been widely studied [5,13,14,15,16,17,18,19], only few studies have investigated its metabolic characteristics [15,20,21]. A previous study identified four hydroxylated and four glucuronide conjugates of loxoprofen after oral administration to monkey [21]. The in vivo metabolites of loxoprofen had been profiled in the plasma, urine, and skin of rats [15]. However, to the best of our knowledge, no detailed studies on the in vitro metabolism of loxoprofen in the human liver microsomes (HLMs) have been documented (Figure 1a).

In the present study, we investigated the loxoprofen metabolism in human, mouse, dog, rat, and monkey liver microsomes so as to investigate any possible in vitro metabolite formation of loxoprofen by microsomal enzymes. We also identified the possible cytochrome P450 (CYP) and UDP-glucuronosyltransferase (UGT) isoforms involved in the biotransformation of loxoprofen in HLMs. We identified four microsomal enzymes that produce metabolites of loxoprofen, including the active trans-alcohol metabolite. We found that CYP3A4 and CYP3A5 are the major CYP isoforms involved in the hydroxylation of loxoprofen. Similarly, we identified two glucuronide metabolites of loxoprofen that are mainly produced by UGT2B7 and UGT1A6.

## 2. Materials and Methods

### 2.1. Materials

Loxoprofen was purchased from Tokyo Chemicals Industry (Tokyo, Japan). Trans-OH and Cis-OH loxoprofen (purities 96.5% and 97.9%, respectively) were chemically synthesized from loxoprofen [22]. The β-reduced nicotinamide adenine dinucleotide phosphate (β-NADPH) regeneration system (NGS) was purchased from Promega Corp. (Madison, WI, USA). Pooled HLMs (mixed gender) were purchased from Sekisui Xeno Tech, LLC (Kansas City, MO, USA). Mixed Gender Corning UltraPool 150-donor liver cytosols; mouse, rat, dog, and monkey liver microsomes (RLM, MLM, DLM, and MoLM, respectively); and purified human recombinant cDNA encoding CYPs and UGTs were purchased from Corning Gentest (Woburn, MA, USA). Alamethicin and Uridine 5′-diphosphoglucuronic acid trisodium (UDPGA) were obtained from Sigma-Aldrich (St. Louis, MO, USA). Mass spectrometry (MS) grade water and acetonitrile (ACN) were acquired from Fischer Scientific (Pittsburgh, PA, USA).

### 2.2. Metabolic Stabilities in Human Liver Microsomes and the Cytosols

To properly understand the metabolism of loxoprofen, its metabolic stability was compared between human liver cytosols (HLCs) and HLMs in the presence of NGS and UDPGA. The phase I metabolic stability was performed by incubating loxoprofen (5 µM) with 1 mg/mL of HLC or HLMs in 0.1 M phosphate buffer (pH 7.4) at 37 °C, with the addition of NGS to give a total reaction volume of 100 µL. The reaction was terminated at 0, 20, 40, and 60 min time points by the addition of ice cold 100% ACN, containing tolbutamide as an internal standard. The sample was vortexed and kept on ice for complete protein denaturation. Following centrifugation for 10 min at 13,000 × *g* at 4 °C, the supernatant was transferred to high performance liquid chromatography (HPLC) vials and analyzed using a TSQ vantage mass spectrometer (Thermo Fisher Scientific, Waltham, MA, USA).

Similarly, for the Phase II metabolic stability of loxoprofen, 1 mg/mL of HLM was treated with 25 µg/mL of alamethicin and kept on ice for 20 min. Then, 5 µM (final concentration) of loxoprofen was added to the reaction mixture and pre-incubated for 5 min at 37 °C in the presence of NGS. The reaction was finally initiated by the addition of cofactor UDPGA (5 mM), and incubated at 37 °C. The samples were then subjected to the same post-reaction procedure as that for Phase I metabolic stability.

### 2.3. Biotransformation of Loxoprofen in Microsomes and the Cytosols

To perform the metabolite profiling, loxoprofen (20 µM) was incubated with 1 mg/mL of HLC or HLM, MLM, RLM, DLM, and MoLM, with the addition of 0.1 M of phosphate buffer (pH 7.4), in the presence or absence of NGS at 37 °C for 60 min. The final reaction volume was 200 µL and the reaction was terminated by adding 400 µL of 100% ACN. The samples were centrifuged at 13,000× *g* at 4 °C for 10 min, and the supernatants (550 µL) were taken and dried under vacuum using a Labonco speed-vac concentrator (Kansas City, MO, USA) at 35 °C. The dried sample was stored at −80 °C until use. The dried samples were reconstituted with 20% ACN (MS grade) and vortexed for at least 10 min. The samples were then centrifuged and 10 µL of the supernatant was analyzed using high-resolution mass spectrometry coupled with liquid chromatography (LC-HR/MS), and the metabolites were identified by studying the chromatographs and spectrums of the *m*/*z* of all of the possible Phase I metabolites.

For the UGT-mediated metabolism of loxoprofen, 1 mg/mL of a liver preparation was treated with 25 µg/mL (final concentration) of alamethicin for 20 min. Then, 20 µM of loxoprofen and 0.1 M phosphate buffer (pH 7.4) were added and pre-incubated for 5 min after addition of NGS. The reaction was started by incubating the reaction mixture at 37 °C in presence or absence of 5 mM UDPGA in a reaction volume of 200 µL. After 60 min, the reaction was quenched with 400 µL of 100% MS grade ACN, vortexed, and centrifuged at 13,000 × *g* at 4 °C for 10 min. The supernatant (550 µL) was removed and dried in a speed-vac at 35 °C. The dried samples were reconstituted with 20% ACN (MS grade) and underwent similar procedures as mentioned for the Phase I metabolism.

### 2.4. Metabolism of Loxoprofen in Chemically-Induced Microsomes and CYP Enzyme Inhibitors

To better understand the CYP-mediated metabolism of loxoprofen, we prepared microsomes enriched with specific CYPs after a selective chemical induction [23]. Four selective inducers were administered to male Sprague-Dawley rats, 3-methylcholanthrene (3-MC) for CYP1A, phenobarbital for CYP2B, dexamethasone for CYP3A, and acetone for CYP2E (Appendix A). Loxoprofen (20 µM) was incubated with 1 mg/mL of enriched microsomes, with the addition of 0.1 M phosphate buffer (pH 7.4) in the presence of NGS at 37 °C for 60 min. The effect of SKF-525A (non-selective CYP inhibitor) and ketoconazole (CYP3A4/5 selective inhibitor) on the metabolism of loxoprofen was investigated in the pooled HLMs. The incubations were performed with an inhibitor (25 and 50 µM for SKF-525A, 1 and 10 µM for ketoconazole, respectively), HLMs (1 mg/mL), and loxoprofen (20 μM) in 0.1 M phosphate buffer (pH 7.4), in the presence of NGS at 37 °C for 60 min.

### 2.5. Recombinant cDNA-Expressed CYPs and UGTs Metabolism of Loxoprofen

To identify the metabolic enzymes for loxoprofen, loxoprofen was incubated with purified CYP and UGT isoforms. For the CYP metabolism study, 5 µM of loxoprofen was incubated with 5 pmol of 10 CYP isoforms (CYP1A1, 1A2, 2B6, 2C8, 2C9, 2C19, 3A4, 3A5, 2D6, and 2E1) at 37 °C for 60 min. The reaction volume was 200 µL. The reaction was stopped by the addition of 400 µL of 100% ACN and centrifugation at 13,000 × *g* for 10 min. The supernatant (550 µL) was dried, reconstituted with 20% ACN, and analyzed in LC-MS.

To detect the glucuronide metabolites of loxoprofen, 5 µM of loxoprofen, Trans-OH, or Cis-OH loxoprofen was incubated with 0.1 mg/mL of five purified UGT isoforms (UGT1A1, 1A3, 1A4, 1A6, 1A9, and 2B7) in presence of 5 mM of UDPGA with a reaction volume of 200 µL at 37 °C for 60 min. The reaction was stopped by 100% ACN and then processed according to the procedure mentioned in the previous paragraph.

### 2.6. Instrument

The LC-MS/MS system consisted of the Thermo Scientific™ Dionex™ Ultimate™ 3000 UHPLC system (Dionex Softron GmbH, Germering, Germany), equipped with an HPG-3200SD Standard binary pump, WPS 3000 TRS analytical autosampler, and a TCC-3000 SD Column compartment. The LC system was coupled with a high-resolution mass spectrometer, the Thermo Scientific™ Q Exactive™ Focus quadrupole-Orbitrap MS (Thermo Fisher Scientific, Bremen, Germany). A heated electrospray ionization source II (HESI-II) probe was used as the ion generator, with nitrogen used as the auxiliary, sheath, and sweep gas. The mass spectrometer was calibrated in both positive and negative mode using a Pierce™ LTQ Velos ESI Positive Ion Calibration Solution and Pierce™ ESI Negative Ion Calibration Solution (Pierce Biotechnology, Rockford, IL, USA) respectively to ensure the mass accuracy of the mass spectrometer. The mass spectrometer was operated in negative ion mode, with sheath gas, and auxiliary gas set to 35 and 12 aux units, respectively. The other parameters were set as follows: the spray voltage to 2.5 KV, capillary temperature to 320 °C, S-lens RF level to 50, and aux gas heater temperature to 200 °C. For the metabolic profiling, a reverse-phase liquid chromatography column (Kinetex^®^ C18 column (150 mm × 2.1 mm, 2.6-μm, Phenomenex, Torrance, CA, USA) was employed at 40 °C. The mobile phase consisted of 100% MS grade water with 0.1% formic acid as solvent A, and 100% MS ACN with 0.1% formic acid as solvent B. The gradient elution was used at a flow rate of 250 µL/min for adequate compound separation, starting with 10% of solvent B for 0.5 min, gradually increasing to 50% in 21.0 min, again increased to 95% over a minute, and kept constant for 3 min before equilibrating the column with 10% solvent B for 5 min.

## 3. Results

### 3.1. Microsomal Metabolism of Loxoprofen

To determine the importance of the microsomal metabolism of loxoprofen, the metabolic stability test was performed in HLC or HLMs in the presence of NGS or UDPGA (Figure 1b). The comparison of the Phase I metabolic stability of the 5 µM loxoprofen between HLC and HLMs showed a marked decrease in the stability of loxoprofen in HLMs compared to that in HLC. Moreover, the metabolic stability of loxoprofen decreased further when it was incubated with UDPGA to stimulate glucuronide conjugation (Phase 2 metabolism). Although the major bioactivation of loxoprofen is mediated by cytosolic carbonyl reductase enzymes, our metabolic stability study suggested that loxoprofen undergoes extensive metabolism in HLMs. 

**Figure 2:**
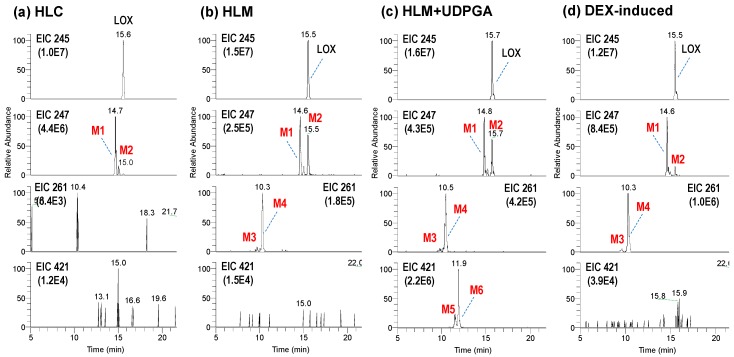
The extracted ion chromatograph (EIC); DEX, dexamethasone EIC of loxoprofen and its metabolites in human liver cytosols (HLC) (**a**) and human liver microsomes (HLM) (**b**) in presence of β-NADPH regeneration system (NGS), HLMs in presence of NGS and UDPGA (**c**), and dexamethasone (DEX)-induced rat liver microsomes (**d**) in the presence of NGS.

The metabolic stability is linked to the type of metabolites produced in the cytosols and microsomes. In fact, more diverse metabolites were produced in the microsomes compared to those in the cytosols. In Figure 2, the representative extraction chromatograms obtained following the incubation of loxoprofen in HLC and HLMs are shown. Six different metabolites of loxoprofen were identified, including two alcohol metabolites (M1 and M2), two mono-hydroxylated metabolites (M3 and M4), and two conjugates with glucuronide (M5 and M6). In particular, the metabolism of loxoprofen in HLC in presence of NGS produced only the two alcohol metabolites (reduction of ketone to alcohol); M1, the active Trans-OH metabolite, C_15_H_19_O_3_ (*m*/*z* 247.1335) at a retention time of 14.6 min, and M2, the cis-alcohol (Cis-OH) metabolite, C_15_H_19_O_3_ (*m*/*z* 247.1334) at a retention time of 15.0 min (Figure 2a). The bioactivation of loxoprofen to its active (trans-alcohol) metabolite, M1, was about five times higher than that of its cis isoform.

Two peaks of hydroxylated metabolites, M3 and M4 (*m*/*z* 261.1129 and 261.1128, C_15_H_17_O_4_) were observed at 9.8 and 10.3 mins, respectively, after the incubation of HLM in the presence of NGS. The yields of alcohol metabolites and hydroxylated metabolites were similar in the control HLMs (Figure 2b). In the presence of UDPGA, two glucuronide metabolites, M5 and M6 (*m*/*z* 421.1498 and 421.1500, C_21_H_25_O_9_) at retention times of 11.4 and 11.7 min, respectively, were generated (Figure 2d). M5 and M6 were hypothesized to be the acyl glucuronides of loxoprofen. 

**Figure 3:**
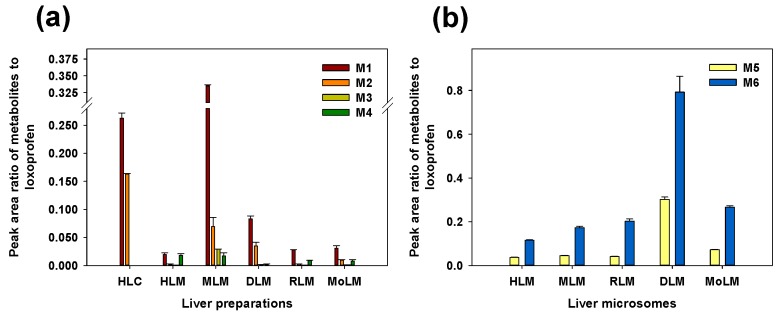
Formation of Phase 1 metabolites (**a**) and Phase 2 conjugates (M5 and M6) (**b**) in the liver microsomes of five mammalian species. The data are represented as mean ± S.E of area ratio of metabolites to loxoprofen of triplicate samples. HLC—human liver cytosols; HLM—human liver microsomes; MLM—mouse liver microsomes; DLM—dog liver microsomes; RLM—rat liver microsomes; MoLM—monkey liver microsomes.

The in vitro metabolic profile of loxoprofen in MLM, DLM, RLM, and MoLM were found to be similar to that obtained in the HLMs (Figure 3a). A higher Phase I metabolism was observed in MLMs compared to the microsomes from the other species, while a higher glucuronidation was observed in the DLMs compared to the microsomes from the other species (Figure 3b).

### 3.2. Structure Elucidation of Microsomal Metabolites In Vitro

Full MS and CID MS/MS scans were performed in negative ion mode for the characterization of metabolites structures using high resolution mass spectrometry coupled with ultra-high performance liquid chromatography (LC-HRMS). The structure of the metabolites was confirmed from the MS/MS fragmentation pattern with exact mass measurement of the precursor and product ions having mass accuracy <5 ppm. 

**Figure A1:**
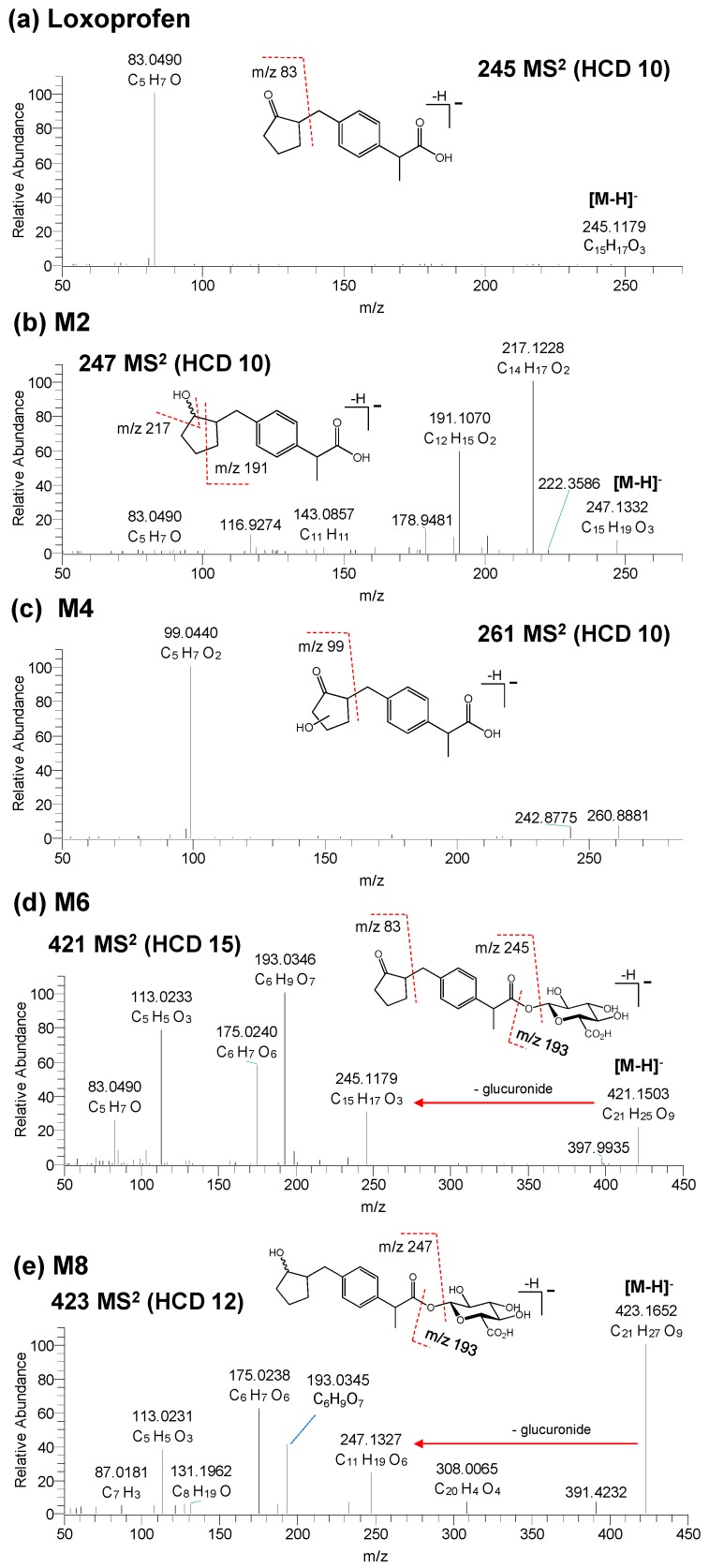
Collision-induced dissociation (CID) spectra of de-protonated loxoprofen (**a**), M2 (**b**), M4 (**c**), M6 (**d**), and M8 (**e**).

M1 and M2 were identified as Trans-OH and Cis-OH metabolites, respectively. The stereochemistry of two alcoholic metabolites were confirmed by comparing the MS^2^ fragmentation pattern and chromatographic retention time of chemically synthesized standard compounds (data not shown). The major product ions at *m*/*z* 217.1228 (C_14_H_17_O_2_) and 191.1070 (C_12_H_15_O_2_) showed that fragmentation occurred at the cyclopentanol ring of the loxoprofen alcohol metabolites. MS/MS fragmentation of M3 and M4 produced a major ion (C_5_H_7_O_2_) of *m*/*z* 99.0440 (C_5_H_7_O_2_), which suggested that the hydroxylation occurred at the cyclopentanone ring of loxoprofen. M5 and M6 were hypothesized to be the acyl glucuronides of loxoprofen, which were supported by major product ions at *m*/*z* 245.1179 (C_15_H_17_O_3_), 193.0346 (C_6_H_9_O_7_), and 83.0490 (C_5_H_7_O) (Figure A1).

### 3.3. Reaction Phenotyping for Phase 1 and Phase 2

**Figure 4:**
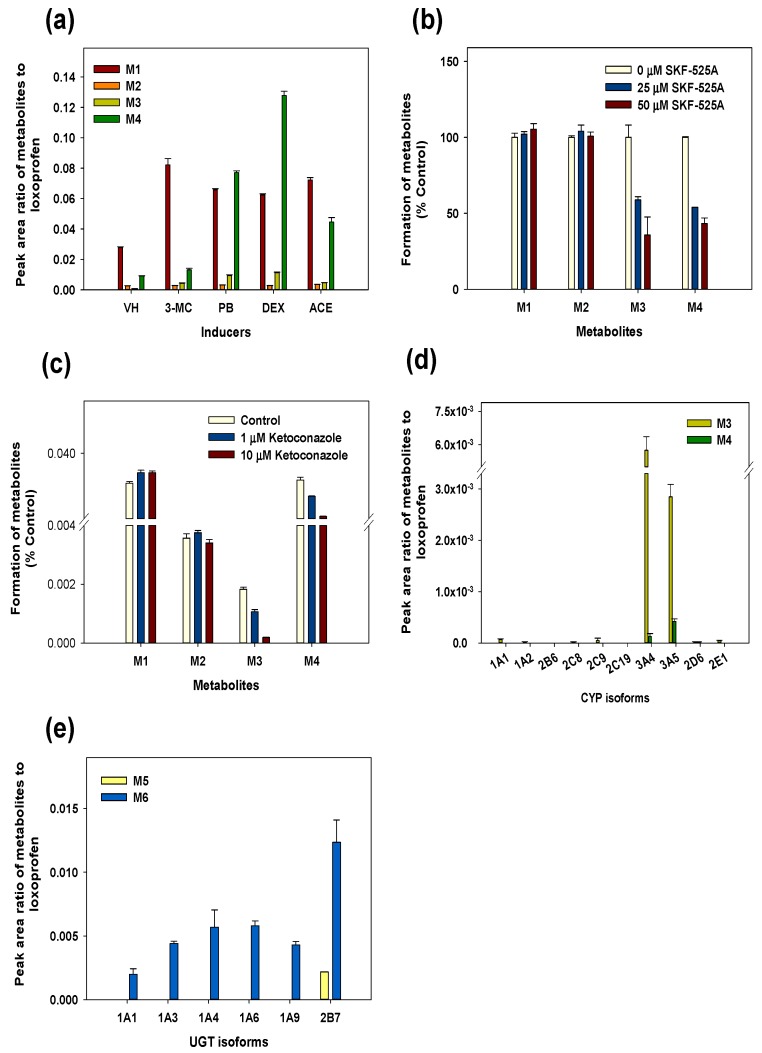
Formation of metabolites expressed as peak area ratio of metabolites to loxoprofen in chemically-induced rat liver microsomes (**a**). Effect of the non-selective cytochrome P450 (CYP) inhibitor, SKF 525-A (25 and 50 µM) (**b**), and the CYP3A4 selective inhibitor, ketoconazole (1 and 10 µM) (**c**), on the metabolism of loxoprofen in human liver microsomes after incubation with 20 µM loxoprofen in presence of a β-NAPDH regeneration system (NGS) for 60 min at 37 °C. The formation of Phase 1 metabolites and Phase 2 conjugates after the incubation of 5 µM loxoprofen with human recombinant cDNA-expressed CYP isoforms in the presence of NGS (**d**), and UDP-glucuronosyltransferases (UGTs) isoforms in the presence of NGS and uridine 5′-diphosphoglucuronic acid (**e**) at 37 °C for 60 min. The data are represented as mean ± standard error (S.E.) of the triplicate samples. VH—vehicle control (untreated rat liver microsomes); 3-MC—3-methylcholanthrene; PB—phenobarbital; DEX—dexamethasone; ACE—acetone.

To investigate the possible CYP isozymes involved in the loxoprofen metabolism, the products formed during the incubation of loxoprofen with the control, 3-methylcholanthrene, phenobarbital (PB), dexamethasone (DEX), and acetone-induced RLMs were determined and displayed in Figure 4a. The production of M4 was increased in the DEX-induced RLMs by 15-fold compared to that in the uninduced control microsomes. Metabolite M3 was produced in PB and DEX-induced microsomes at higher levels compared to that in the uninduced microsomes. We also observed an increase in M3 and M4 formation in the DEX-induced RLMs compared to with that in the control HLMs (Figure 4a). The formation of M3 and M4 was significantly inhibited by SKF-525A, a non-specific CYP inhibitor, indicating that the Phase 1 metabolism of loxoprofen depended on CYP (Figure 4b) [24]. M1 (Trans-OH form) and M2 (Cis-OH form) are products of a reductase; therefore, the formation of M1 and M2 were not inhibited by SKF-525A treatment.

Additionally, the metabolism of loxoprofen dependent on the CYP3A subfamily was confirmed by incubation with a selective CYP3A inhibitor in HLMs (Figure 4c). Ketoconazole, a selective inhibitor of CYP3A4, strongly inhibited the formation of M3 and M4 in a concentration-dependent manner, whereas the formation of M1 and M2 was not affected by ketoconazole. The selective formation of M3 and M4 were finally confirmed by incubation with ten different CYP isoforms (CYP1A1, 1A2, 2B6, 2C8, 2C9, 2C19, 3A4, 3A5, 2D6, and 2E1) (Figure 4d). M3 and M4 were predominantly generated by CYP3A4 and 3A5 in HLMs. 

Following the incubation of loxoprofen with six UGT enzyme isoforms (UGT1A1, 1A3, 1A4, 1A6, 1A9, and 2B7), we found that the UGT2B7 isoform predominantly mediated the glucuronidation of loxoprofen to its glucuronide metabolites, M5 and M6. M5 was detected only in the UGT2B7 isoform, whereas UGT1A4, 1A6, 1A3, and 1A9 contributed to the formation of M6 to a minor extent.

**Figure 5:**
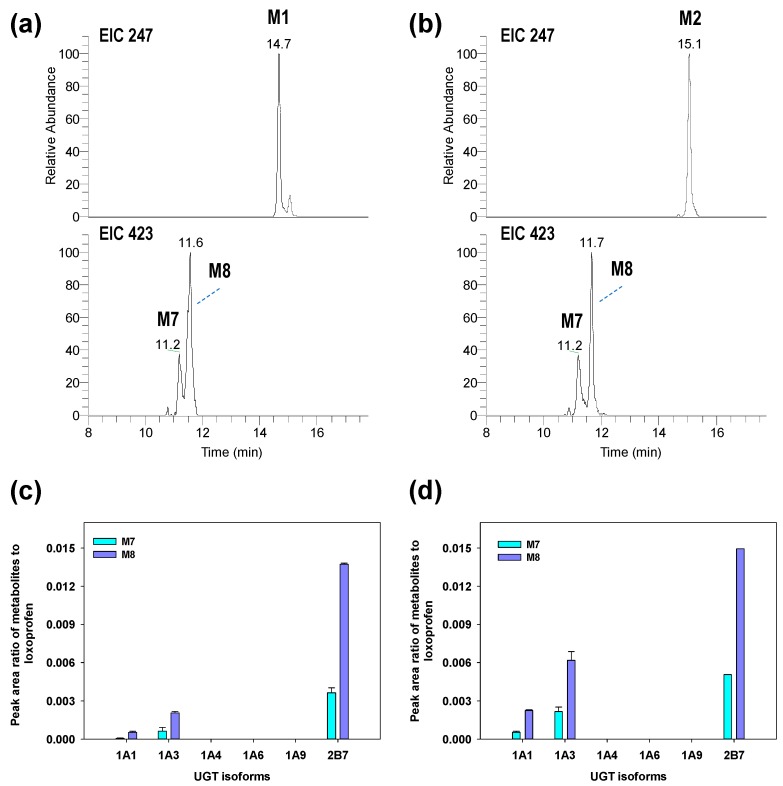
EIC of chemically synthesized Trans-OH (**a**) and Cis-OH standard (**b**) and their glucuronyl conjugates in human recombinant cDNA-expressed UGT2B7 on a Kinetex^®^ C-18 column. The formation of glucuronide metabolites, M7 and M8, expressed as a peak area ratio of metabolites to loxoprofen, after incubating 5 µM of chemically synthesized standards of Trans-OH (**c**) and Cis-OH (**d**) in presence of uridine 5′-diphosphoglucuronic acid in six different UDP-glucuronosyltransferase (UGT) isoforms (UGT1A1, 1A3, 1A4, 1A6, 1A9, and 2B7) at 37 °C for 60 min. The data is represented as mean ± S.E of the duplicate samples.

**Figure 6:**
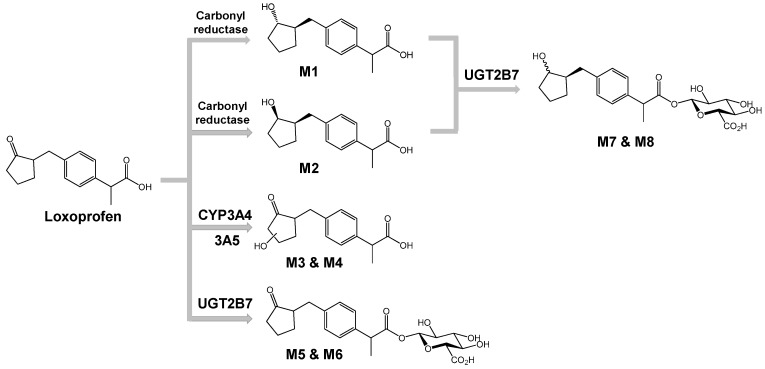
Metabolic pathway of loxoprofen in human liver microsomes.

The previous in vivo study on the metabolism of loxoprofen reported the formation of two glucuronide conjugates of the alcohol metabolites of loxoprofen (Lox-OH-glucuronide) in monkey, mouse and dog urine (15, 16). Although we were unable to detect these metabolites after the incubation of loxoprofen with purified UGT enzyme isoforms, we incubated chemically synthesized Trans-OH or Cis-OH standard with six UGT isoforms (UGT1A1, 1A3, 1A4, 1A6, 1A9, and 2B7) in the presence of NGS and UDPGA, which yielded two Lox-OH-glucuronides, M7 and M8, of *m*/*z* 423.1652 primarily by UGT2B7 isoform at retention time of 11.2 and 11.7 mins (Figure 5). The CID MS/MS fragmentation of M7 and M8 resulted in product ions at *m*/*z* 247.1327 (C_15_H_19_O_3_, after loss of a glucuronide ion) and 193.0345 (C_6_H_9_O_7_) (Figure A1). From this fragmentation pattern, we proposed that M7 and M8 are the acyl glucuronide conjugates of the alcohol metabolites of loxoprofen. The final postulated metabolic pathway was shown in Figure 6.

## 4. Discussion

Loxoprofen is a popular drug of choice for musculoskeletal pain relief in many East-Asian countries. However, until now, the microsomal metabolic profile of loxoprofen was unknown. The active metabolite of loxoprofen, the Trans-OH form, exhibits anti-inflammatory properties by inhibiting cyclooxygenase enzymes. However, not only is the non-selective inhibition of cyclooxygenase enzymes of loxoprofen associated with adverse effects, but also, other ADRs of loxoprofen have been reported [9,10]. To prevent the toxicity caused by the drug–drug interaction, the metabolic profiling of loxoprofen should be clarified. In the present study, we investigated the metabolic pathway of loxoprofen in the liver microsomes from five mammalian species and identified the enzymes involved in the metabolism of loxoprofen in HLMs. 

In the liver microsomes of the five mammalian species, we identified two hydroxylated metabolites (M3 and M4) and two glucuronide metabolites (M5 and M6). A comparison of the metabolic activity among five mammalian species revealed a higher Phase I metabolism in the mouse liver microsomes compared with that in the other species, probably reflecting its higher CYP content (pmol/mg protein) [25]. The dog liver microsomes produced higher levels of the glucuronide metabolites compared to those produced by the other species. Previous studies showed that the UGT1A1 and UGT2B7 substrates were highly metabolized by several-fold to their glucuronide metabolites in the dog liver compared with that in the human liver [26,27].

Incubation with chemically induced microsomes, a CYP3A4/5 selective inhibitor, and human recombinant cDNA-expressed CYPs (Figure 3a,d,e), showed that CYP3A4 and CYP3A5 are the primary CYP isoforms responsible for the microsomal hydroxylation of loxoprofen. CYP3A is the major CYP subfamily involved in the metabolism of more than 60% of drugs metabolized by CYP enzymes [28]. Many toxicities associated with modulation of CYP3A activity have been reported [29,30]. The metabolism of tacrolimus by the CYP3A subfamily in human liver microsomes was inhibited by loxoprofen to a lesser extent [31]. From previous studies, it is found that the polymorphism of the CYP3A subfamily either has no effect or shows a decrease in the expression or activity of the CYP3A subfamily. However, there have been a number of drugs that are known to induce CYP3A4 isoforms in humans, such as rifampicin, phenytoin, St. John’s wort, and carbamazepine [32]. Previous study showed that the induction of the CYP3A4 isoform by rifampicin increased the acetaminophen induced toxicity [33]. The induction of the CYP3A4 isoform might alter the metabolic pathway of loxoprofen, leading to the reduced anti-inflammatory activity of loxoprofen, and thus a proper dose adjustment should be made to maintain the optimal therapeutic level of the drug. Therefore, it is important to further investigate the interaction between the loxoprofen and CYP3A activity modulators, which could result in drug–drug interactions.

The structure of loxoprofen contains a carboxylic acid moiety in its aliphatic chain. Drugs with a carboxylic acid moiety have long been associated with idiosyncratic drug toxicity; mainly drug induced liver injury caused by the formation of reactive metabolites like acyl glucuronide, acyl-co-A thioester, and acyl glutathione thioester derivatives [34,35,36]. Generally, glucuronidation is the detoxification and elimination pathway for xenobiotics [37]; however, the electrophilic acyl glucuronide can specifically and covalently bind to proteins like albumin. Neoantigens are formed to govern the immune-mediated reactions caused by the covalent binding of proteins to reactive acyl glucuronides, resulting in the toxicities associated with carboxylic acid containing drugs, which are mainly hypersensitivity reactions [38]. In the present study, we found that the metabolic conversion of loxoprofen to the acyl glucuronide metabolites occurred at a rate similar to that of its active metabolites (M1 and M2). The generated acyl glucuronide might be linked to loxoprofen-induced toxicities, and UGT2B7 was identified as the primary UGT isoform involved in the glucuronidation of loxoprofen and its alcoholic metabolites (M1 and M2) in HLMs. UGT2B7 is a major isoform involved in the metabolism of many NSAID drugs [39,40]. Although the UGT2B7 variants UGT2B7*1 and UGT2B7*2 have been found to be about 10-fold higher in Japanese than in Caucasians, this UGT2B7 polymorphism showed no significant alteration in the enzyme activity. The concomitant administration of drugs inducing UGT2B7 isoform, like phenobarbital and rifampicin, with loxoprofen, could lead to deleterious adverse effects from the increase in acyl glucuronide formation [41,42]. Owing to the formation of four acyl glucuronides, it is of utmost importance to investigate the toxicity of the acyl glucuronides of loxoprofen. 

## 5. Conclusions

Using LC-HR/MS, four in vitro Phase I metabolites of loxoprofen (two alcohol metabolites and two hydroxylated metabolites) were identified in human, mouse, dog, rat, and monkey liver microsomes. CYP3A4 and 3A5 enzymes were found to be the major enzyme involved in the hydroxylation of loxoprofen, and UGT2B7 was found to be the main enzyme involved in its acyl-conjugation. We postulated that further studies should be carried to assess the potency and toxicity of these identified metabolites of loxoprofen, in order to gain a proper understanding of the mechanism of loxoprofen toxicity.

## Appendix A

### Appendix A.1. Supplemental Methods

#### Animal Treatment and Preparation of Liver Microsomes

Specific pathogen-free male Sprague-Dawley rats (250–280 g) were obtained from Charles River, Korea (Seoul, Korea). The animals were received at 5–6 weeks of age and were acclimated for at least one week. Upon arrival, the animals were randomized and housed as five animals per cage. The animal quarters were strictly maintained at 23 ± 3 °C and 50 ± 10% relative humidity. A 12 h light and dark cycle was used with an intensity of 150–300 Lux. All of the animal procedures were based on the guidelines recommended by the Society of Toxicology (USA) in 1989. For the preparation of enriched microsomes, specific pathogen-free male Sprague-Dawley rats (270–300 g) were pretreated with either 3-methylcholanthrene (3MC, 40 mg/kg, i.p., three days) in corn oil, PB (80 mg/kg, i.p., three days) in saline, dexamethasone (DEX, 50 mg/kg, i.p., three days) in corn oil, or acetone (ACE, 5 mL/kg, p.o., once). Twenty-four hours after the last dose, or two days after the last dose in the case of ACE, the enriched liver microsomes were isolated.

#### Instrument for Metabolic Stability

For the metabolic stability, a TSQ vantage triple quadrupole mass spectrometer equipped with a HESI-II Spray source was used, coupled to a Shimadzu Prominence UFLC system (Kyoto, Japan), incorporating a DGU-20A5 degasser, an LC-20AD Pump, an SIL-20A autosampler, and a CTO-20A column oven. The analytes were separated using a Kinetex^®^ C18 column (150 mm × 2.1 mm, 2.6-μm, Phenomenex, Torrance, CA, USA). The mobile phase consisted of 100% ACN with 0.1% formic acid (mobile phase A) and 0.1% aqueous formic acid (mobile phase B) at a flow rate of 0.25 mL/min at 40 °C. The gradient conditions were as follows: 20% of B for 0.5 min, 20–99% of B at 0.5–2 min, 99% of B at 2–7 min, 99–20% of B at 7–7.5 min, and 20% of B at 7.5–12 min. For the qualification and quantitation of loxoprofen and its metabolites, instruments were set for [M−H]^−^ ion. The MS operating conditions were as follows: electrospray ionization in negative mode, spray voltage, 3000 V; capillary temperature, 350 °C; vaporizer temperature, 300 °C; sheath gas pressure, 35 Arb; auxiliary gas pressure, 10 Arb; ion sweep gas pressure, 2.0 Arb. The data were analyzed using the Xcalibur software (Thermo Fisher Scientific Inc., Waltham, MA, USA).

## References

[1] Greig S.L., Garnock-Jones K.P. (2016). Loxoprofen: A review in pain and inflammation. Clin. Drug. Investig..

[2] Asami T., Yamanouchi N., Asami A., Tanaka H., Nogami N. (2013). The effectiveness of patches containing loxoprofen sodium hydrate (lx-p) in the conservative therapy of muscular back pain—Clinical results using the japanese orthopaedic association back pain evaluation questionnaire (joabpeq). Jpn. J. Compr. Rehabil. Sci..

[3] Waikakul S., Soparat K. (1995). Effectiveness and safety of loxoprofen compared with naproxen in nonsurgical low back pain. Clin. Drug. Investig..

[4] Nagashima H., Tanaka Y., Watanabe H., Hayashi R., Kawada K. (1984). Optical inversion of (2R)-to (2S)-isomers of 2-[4-(2-administration oxocyclopentylmethyl)-phenyl] propionic acid (loxoprofen), a new anti-inflammatory agent, and its monohydroxy metabolites in the rat. Chem. Pharm. Bull..

[5] Koo T.S., Kim D.H., Ahn S.H., Kim K.P., Kim I.W., Seo S.Y., Suh Y.G., Kim D.D., Shim C.K., Chung S.J. (2005). Comparison of pharmacokinetics of loxoprofen and its active metabolites after an intravenous, intramuscular, and oral of loxoprofen in rats: Evidence for extrahepatic metabolism. J. Pharm. Sci..

[6] Sakamoto C., Kawai T., Nakamura S., Sugioka T., Tabira J. (2013). Comparison of gastroduodenal ulcer incidence in healthy japanese subjects taking celecoxib or loxoprofen evaluated by endoscopy: A placebo-controlled, double-blind 2-week study. Aliment. Pharmacol. Ther..

[7] Sekiguchi H., Inoue G., Nakazawa T., Imura T., Saito W., Uchida K., Miyagi M., Takahira N., Takaso M. (2015). Loxoprofen sodium and celecoxib for postoperative pain in patients after spinal surgery: A randomized comparative study. J. Orthop. Sci..

[8] Kaniwa N., Ueta M., Nakamura R., Okamoto-Uchida Y., Sugiyama E., Maekawa K., Takahashi Y., Furuya H., Yagami A., Matsukura S. (2015). Drugs causing severe ocular surface involvements in japanese patients with stevensejohnson syndrome/toxic epidermal necrolysis. Allergol. Int..

[9] Ueharaguchi Y. (2011). Loxoprofen/piperacillin acute generalised exanthematous pustulosis. Reactions.

[10] Yue Z., Shi J., Jiang P., Sun H. (2014). Acute kidney injury during concomitant use of valacyclovir and loxoprofen: Detecting drug–drug interactions in a spontaneous reporting system. Pharmacoepidemiol. Drug Saf..

[11] Taniguchi Y., Deguchi Y., Noda K. (1996). Interaction between enoxacin, a new antimicrobial, and nimesulide, a new non-steroidal anti-inflammatory agent in mice. Inflamm. Res..

[12] Shibata K., Akagi Y., Nozawa N., Shimomura H., Aoyama T. (2017). Influence of nonsteroidal anti-inflammatory drugs on aspirin’s antiplatelet effects and suggestion of the most suitable time for administration of both agents without resulting in interaction. J. Pharm. Health Care Sci..

[13] Yamakawa N., Suemasu S., Watanabe H., Tahara K., Tanaka K.-I., Okamoto Y., Ohtsuka M., Maruyama T., Mizushima T. (2013). Comparison of pharmacokinetics between loxoprofen and its derivative with lower ulcerogenic activity, fluoro-loxoprofen. Drug Metab. Pharmacokinet..

[14] Lee H.W., Ji H.Y., Sohn D.H., Kim S.M., Lee Y.B., Lee H.S. (2009). Liquid chromatography-tandem mass spectrometry method of loxoprofen in human plasma. Biomed. Chromatogr..

[15] Sawamura R., Kazui M., Kurihara A., Izumi T. (2014). Absorption, distribution, metabolism and excretion of loxoprofen after dermal application of loxoprofen gel to rats. Xenobiotica.

[16] Sawamura R., Sakurai H., Wada N., Nishiya Y., Honda T., Kazui M., Kurihara A., Shinagawa A., Izumi T. (2015). Bioactivation of loxoprofen to a pharmacologically active metabolite and its disposition kinetics in human skin. Biopharm. Drug Dispos..

[17] Jhee O.H., Lee M.H., Shaw L.M., Lee S.E., Park J.H., Kang J.S. (2007). Pharmacokinetics and bioequivalence study of two brands of loxoprofen tablets in healthy volunteers. Arzneimittelforschung.

[18] Cho H.-Y., Park C.-H., Lee Y.-B. (2006). Direct and simultaneous analysis of loxoprofen and its diastereometric alcohol metabolites in human serum by on-line column switching liquid chromatography and its application to a pharmacokinetic study. J. Chromatogr. B Analyt. Technol. Biomed. Life Sci..

[19] Kanazawa H., Tsubayashi A., Nagata Y., Matsushima Y., Mori C., Kizu J., Higaki M. (2002). Stereospecific analysis of loxoprofen in plasma by chiral column liquid chromatography with a circular dichroism-based detector. J. Chromatogr. A.

[20] Kim I.-W., Chung S.-J., Shim C.-K. (2002). Altered metabolism of orally administered loxoprofen in human subjects after an oral administration of loxoprofen for three consecutive days followed by a seven-day washout. J. Pharm. Sci..

[21] Tanaka Y., Nishikawa Y., Hayashi R. (1983). Species differences in metabolism of sodium 2-[4-(2-oxocyclopentylmethyl)-phenyl] propionate dihydrate (loxoprofen sodium), a new anti-inflammatory agent. Chem. Pharm. Bull..

[22] Naruto S., Terada A. (1983). Synthesis of the 8 possible optically-active isomers of 2-[4-(2-hydroxycyclopentylmethyl) phenyl] propionic acid. Chem. Pharm. Bull..

[23] Kim D.O., Lee S.K., Jeon T.W., Jin C.H., Hyun S.H., Kim E.J., Moon G.I., Kim J.A., Lee E.S., Lee B.M. (2005). Role of metabolism in parathion-induced hepatotoxicity and immunotoxicity. J. Toxicol. Environ. Health A.

[24] Emoto C., Murase S., Sawada Y., Jones B.C., Iwasaki K. (2003). In vitro inhibitory effect of 1-aminobenzotriazole on drug oxidations catalyzed by human cytochrome P450 enzymes: A comparison with SKF-525A and ketoconazole. Drug Metab. Pharmacokinet..

[25] Pasanen M. (2014). Species differences in CYP enzymes. Monografías de la Real Academia Nacional de Farmacia.

[26] Sharer J.E., Shipley L.A., Vandenbranden M.R., Binkley S.N., Wrighton S.A. (1995). Comparisons of phase i and phase ii in vitro hepatic enzyme activities of human, dog, rhesus monkey, and cynomolgus monkey. Drug Metab. Dispos.

[27] Soars M.G., Riley R.J., Findlay K.A., Coffey M.J., Burchell B. (2001). Evidence for significant differences in microsomal drug glucuronidation by canine and human liver and kidney. Drug Metab. Dispos.

[28] Gibson G., Plant N., Swales K., Ayrton A., El-Sankary W. (2002). Receptor-dependent transcriptional activation of cytochrome P4503A genes: Induction mechanisms, species differences and interindividual variation in man. Xenobiotica.

[29] Honig P.K., Wortham D.C., Zamani K., Conner D.P., Mullin J.C., Cantilena L.R. (1993). Terfenadine-ketoconazole interaction. JAMA.

[30] Huang S.M., Strong J.M., Zhang L., Reynolds K.S., Nallani S., Temple R., Abraham S., Habet S.A., Baweja R.K., Burckart G.J. (2008). New era in drug interaction evaluation: Us food and drug administration update on CYP enzymes, transporters, and the guidance process. J. Clin. Pharmacol..

[31] Iwasaki K., Matsuda H., Nagase K., Shiraga T., Tokuma Y., Uchida K. (1993). Effects of twenty-three drugs on the metabolism of FK506 by human liver microsomes. Res. Commun. Chem. Pathol. Pharmacol.

[32] Klaassen C. (2013). Casarett & Doull’s Toxicology: The Basic Science of Poisons.

[33] Cheng J., Ma X., Krausz K.W., Idle J.R., Gonzalez F.J. (2009). Rifampicin-activated human PXR and CYP3A4 induction enhance acetaminophen-induced toxicity. Drug Metab. Dispos..

[34] Darnell M., Weidolf L. (2013). Metabolism of xenobiotic carboxylic acids: Focus on coenzyme a conjugation, reactivity, and interference with lipid metabolism. Chem. Res. Toxicol..

[35] Lassila T., Hokkanen J., Aatsinki S.M., Mattila S., Turpeinen M., Tolonen A. (2015). Toxicity of carboxylic acid-containing drugs: The role of acyl migration and coa conjugation investigated. Chem. Res. Toxicol..

[36] Skonberg C., Olsen J., Madsen K.G., Hansen S.H., Grillo M.P. (2008). Metabolic activation of carboxylic acids. Expert Opin. Drug Metab. Toxicol..

[37] Kroemer H.K., Klotz U. (1992). Glucuronidation of drugs. Clin. Pharmacokinet..

[38] Regan S.L., Maggs J.L., Hammond T.G., Lambert C., Williams D.P., Park B.K. (2010). Acyl glucuronides: The good, the bad and the ugly. Biopharm. Drug Dispos..

[39] Kuehl G.E., Lampe J.W., Potter J.D., Bigler J. (2005). Glucuronidation of nonsteroidal anti-inflammatory drugs: Identifying the enzymes responsible in human liver microsomes. Drug Metab. Dispos..

[40] Jinno N., Tagashira M., Tsurui K., Yamada S. (2014). Contribution of cytochrome P450 and UDT-glucuronosyltransferase to the metabolism of drugs containing carboxylic acid groups: Risk assessment of acylglucuronides using human hepatocytes. Xenobiotica.

[41] Kiang T.K., Ensom M.H., Chang T.K. (2005). UDP-glucuronosyltransferases and clinical drug-drug interactions. Pharmacol. Ther..

[42] Soars M.G., Petullo D.M., Eckstein J.A., Kasper S.C., Wrighton S.A. (2004). An assessment of UDP-glucuronosyltransferase induction using primary human hepatocytes. Drug Metab. Dispos..

